# Earth’s magnetic field remained weak for 40 million years after the Cambrian radiation

**DOI:** 10.1126/sciadv.aeg2325

**Published:** 2026-07-23

**Authors:** Simon J. Lloyd, Andrew J. Biggin, Rebecca Hopkins, Alexander Tully, Brendan Cych, Greig A. Paterson, Ricardo Trindade

**Affiliations:** ^1^ Department of Earth, Ocean and Ecological Sciences, University of Liverpool, Liverpool, UK.; ^2^ University of Sao Paulo, Sao Paulo, Brazil.

## Abstract

The Ediacaran-Cambrian interval marks major environmental change and the emergence of complex life, coinciding with some of the weakest magnetic field strengths in Earth history. It has been proposed that inner core nucleation near this boundary triggered a rapid strengthening of the geodynamo during the early Cambrian, but direct constraints on magnetic field behavior across this interval remain limited. Here, we present new paleointensity results from two Cambrian igneous sequences in Brazil (~525 million years ago) and Nova Scotia (~505 million years ago). Using multiple complementary methods, both records indicate a reversing geomagnetic field that remained weak. Combined with a reanalysis of published data, our results show that weak magnetic field conditions persisted for tens of millions of years after the Cambrian radiation. These findings limit the magnitude and timing of any Cambrian geodynamo strengthening and imply that Earth’s magnetospheric shielding remained strongly suppressed during a key phase of early animal evolution.

## Teaser

Cambrian paleointensity data show that Earth’s magnetic field remained weak long after the Cambrian radiation.

## INTRODUCTION

The Ediacaran-Cambrian interval is central to questions about long-term geodynamo evolution, the timing and expression of inner core nucleation (ICN), and the degree of magnetospheric shielding during early animal diversification (*1*, *2*). It also coincides with major environmental and biological change, including large-scale redox reorganization and the Cambrian radiation. Establishing how geomagnetic field strength evolved across this interval bears directly on models of core evolution, magnetospheric shielding, and the extent to which deep Earth processes may have influenced surface conditions.

Some recent studies propose that a critically diminished late Ediacaran field reflects a weak pre-ICN geodynamo and that ICN near ~550 million years ago (Ma) triggered rapid strengthening during the early Cambrian (*2*–*4*). Existing paleointensity data are consistent with an unusually weak late Ediacaran field (*5*–*9*). A recent hypothesis further proposes that weak geomagnetic shielding promoted atmospheric hydrogen escape and oxygenation, with subsequent strengthening helping to sustain habitable surface conditions (*2*). Other explanations for Ediacaran-Cambrian environmental and biological change invoke nutrient supply, redox evolution, and evolutionary innovation rather than geomagnetic forcing (*10*–*15*). The question of whether a sharp increase in field strength is actually recorded across ~560 to 530 Ma is therefore key to resolve.

If ICN near ~550 Ma produced a sharp and sustained increase in field strength, elevated paleointensities should persist through the tens of millions of years that followed. The available record does not test this clearly. Rocks aged ~420 to 530 Ma remain sparsely sampled, and published estimates are not directly comparable because data treatment differs among studies.

Geodynamo models add further uncertainty. Some numerical studies suggest that inner-core growth could revive a previously weak geodynamo and have therefore been cited in support of an Ediacaran weak field followed by Cambrian strengthening (*16*–*19*). The core’s thermal evolution is, however, highly uncertain and permitting a broad range of possible ICN ages (*16*–*21*). Geodynamo studies also differ in their determinations of whether inner-core growth should produce a sharp paleointensity increase at all (*18*, *19*). The interval immediately after the Cambrian radiation is therefore critical because it determines whether the record supports rapid and sustained recovery or a continued weak paleofield.

Here, we present new paleointensity results from two Cambrian igneous sequences: the Bourinot volcanics of Nova Scotia [~505 Ma; (*22*, *23*)] and the Itabaiana dyke swarm of NE Brazil [~525 Ma; (*24*)]. These localities provide new constraints on geomagnetic field behavior after the proposed ~550 Ma strengthening interval and extend coverage across a key but sparsely sampled part of the early-middle Cambrian record. We then integrate these data with a reassessment of published Cambrian and adjacent Ediacaran estimates using selection criteria optimized for paleointensity experiments (SCOPE), a newly defined set of selection criteria derived from the paleointensity literature and tested against known-field experiments (*25*–*27*) (Supplementary Note 1). A previously published whole-rock estimate from the Chatham-Grenville system also falls within this interval; the technical basis for its treatment in the present synthesis is documented in Supplementary Note 4. Our aims are to establish new Cambrian paleointensity constraints from two well-characterized localities, integrate them into a reassessed synthesis of field-strength estimates across ~600 to 500 Ma, and determine whether that record supports a sharp and sustained strengthening or indicates continued geomagnetic weakness. The implications bear on core evolution, reversal behavior, and magnetospheric shielding during early animal diversification.

## RESULTS

### Study localities and paleomagnetic context

**Fig. 1.**
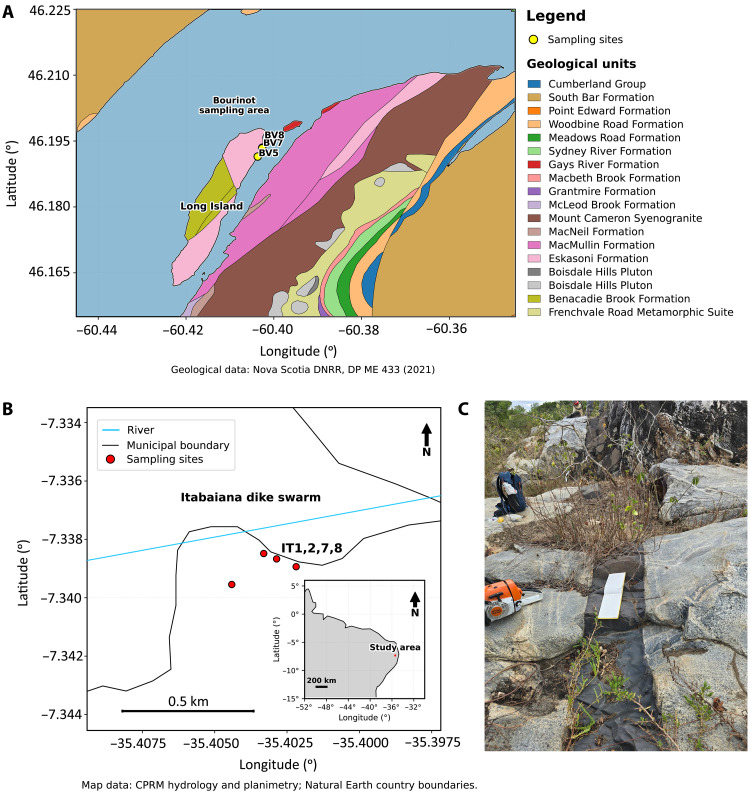
Geological and field context of the Bourinot and Itabaiana sampling sites. (**A**) Simplified geological map of the Bourinot area, Cape Breton Island, Nova Scotia, showing the location of Long Island and the Bourinot sampling sites (BV5, BV7, and BV8). Geological units are shown following the Nova Scotia Department of Natural Resources and Renewables mapping (DP ME 433, 2021), with geological formations. The Bourinot volcanic units are highlighted at the sampling locality. (**B**) Locality map of the Itabaiana dyke swarm (NE Brazil), showing sampling sites (IT1, IT2, IT7, and IT8) relative to regional rivers and administrative boundaries. Inset shows the regional location of the study area within northeastern Brazil. (**C**) Field photograph of a representative Itabaiana dyke (IT1) intruding host granitoid, illustrating dyke geometry and contact relationships. Photo credit: S.J.L., University of Liverpool.

The geological and field context of the Bourinot and Itabaiana sampling localities is shown in Fig. 1.

### Bourinot volcanics

Cambrian volcanic and volcanogenic sedimentary rocks in the Bourinot belt of Cape Breton Island, Nova Scotia, comprise basaltic and more felsic units interbedded with marine to terrestrial sedimentary rocks. Previous paleomagnetic work identified dual-polarity magnetizations, yielded a positive fold test, and interpreted the high-temperature remanence as primary (*23*). Magnetite carries the remanence in unoxidized flows, whereas oxidized flows also contain a higher-temperature Fe-oxide phase acquired during early cooling and exposure. White *et al.* (*22*) reported a U-Pb zircon age of 505 ± 3 Ma for rhyolite flows on Long Island, the sampling locality considered here, and used these data to distinguish the northern Long Island volcanic unit from the southern Middle Cambrian Bourinot Group. We therefore use 505 ± 3 Ma as the age constraint for the sampled Long Island rhyolitic flows.

### Itabaiana dykes

The Itabaiana dyke swarm intrudes Paleoproterozoic gneisses of the Borborema Province in NE Brazil and consists mainly of NW-SE trending olivine-basalt and dolerite dykes, locally crosscut by younger N-S basaltic dykes. Previous paleomagnetic work established that the dykes carry a stable thermal remanent magnetization acquired during emplacement; baked-contact tests on three dykes are positive and dual polarities are recorded (*24*). Remanence is dominantly carried by fine-grained single-domain magnetite with unblocking between ~540° and 560°C (*24*). Two ^40^Ar/^39^Ar plateau ages indicate emplacement at 525 ± 5 Ma and 526 ± 4 Ma (*24*).

### Rock magnetic and petrographic summary

Rock magnetic observations support paleointensity analysis at both localities. In Bourinot, high-temperature susceptibility–temperature (*k*-*T*) and saturation magnetization–temperature (*Ms*-*T*) curves are largely reversible, with only modest heating-cooling divergence at the highest temperatures. Two Curie/Néel temperatures, near ~570° and ~630°C, are consistent with low-Ti titanomagnetite and a higher-temperature Fe-Ti oxide phase, and the thermomagnetic behavior is stable over the fitted intervals (fig. S2A).

In Itabaiana, Curie temperatures cluster near 580°C, and *k*-*T* and *Ms*-*T* behavior indicates a near-stoichiometric magnetite-dominated remanence with generally reversible behavior to at least ~550°C (*24*) (fig. S2, B and C). On a Day plot (*28*), Itabaiana specimens fall toward the top-left part of the distribution (fig. S2C), consistent with fine primary remanence carriers in the single-domain to single-vortex range (*28*, *29*). Microscopy is consistent with these interpretations: Bourinot Fe-Ti oxides show textures compatible with early deuteric oxidation during cooling, whereas Itabaiana opaque phases are dominated by sharp Fe-Ti oxides with no evidence for late low-temperature replacement fabrics at the scale imaged (figs. S2 and S3). Additional rock magnetic, microscopy, and energy-dispersive x-ray spectroscopy (EDX) results are provided in Supplementary Note 2 and fig. S4.

### Cambrian paleointensity results

**Fig. 2.**
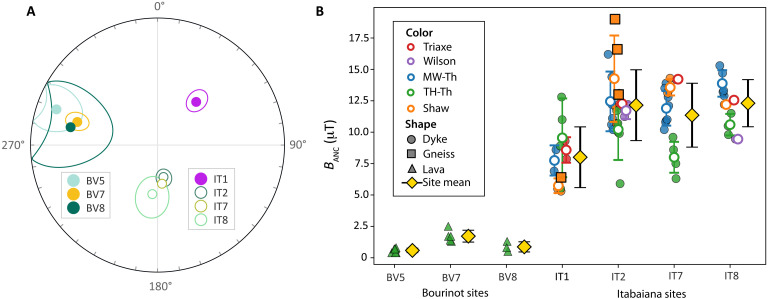
Summary of site-mean results for Bourinot and Itabaiana. (**A**) Paleomagnetic directions; open and closed symbols denote reversed and normal hemisphere, respectively. (**B**) Paleointensity results; individual method means are shown alongside the site overall mean with error bars (± 1σ). MW-Th, microwave Thellier; TH-Th, thermal Thellier. Color denotes the method, and shape denotes the type.

**Fig. 3.**
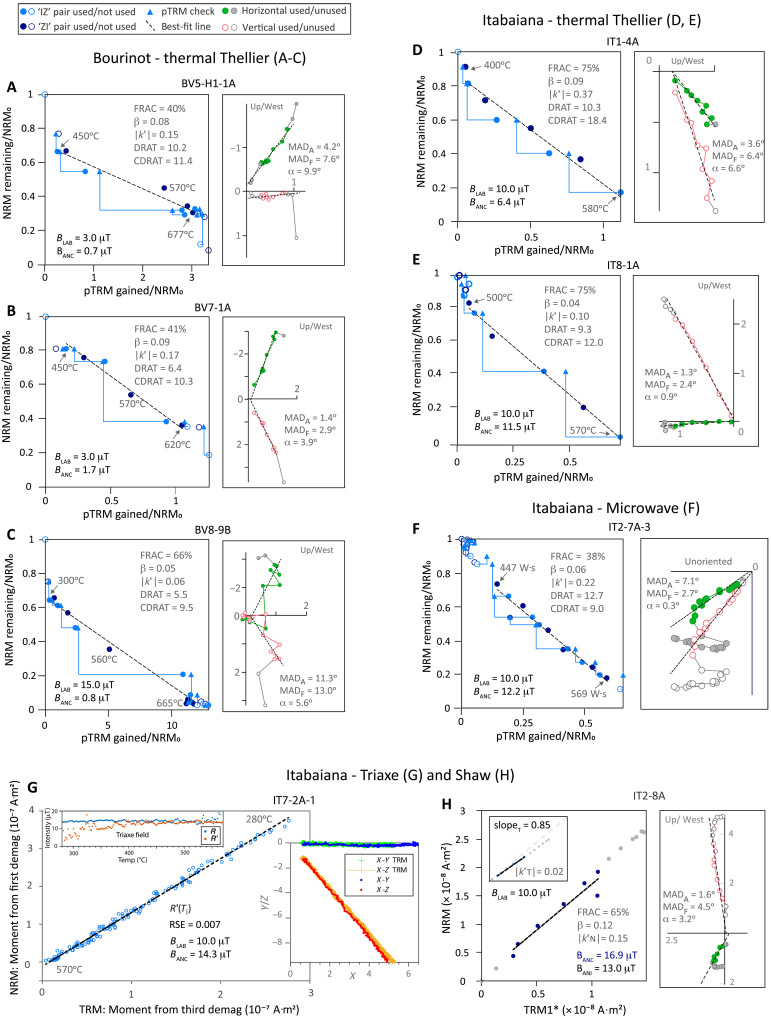
Representative accepted Arai/pseudo-Arai diagrams for Bourinot and Itabaiana. (**A** to **C**) Bourinot thermal Thellier examples. (**D** and **E**) Itabaiana thermal Thellier examples. (**F**) Microwave example. W·s, watt-second. (**G** and **H**) Itabaiana pseudo-Arai examples of Triaxe and Shaw. The latter (H) is a successful result from the baked orthogneiss host rock with a direction matching the original positive baked-contact sample EI26.G1 (*24*). Diagrams produced using paleomagnetism.org (*35*) and paleointensity.org (*36*). FRAC, fraction of remanence used for the best-fit segment; β, relative scatter about the best-fit line; |*k*′|, curvature of the selected Arai segment; DRAT, maximum absolute pTRM-check difference normalized by the length of the best-fit line; CDRAT, cumulative DRAT; α, angle between anchored and free-fit directions; MAD_A_/MAD_F_, maximum angular deviation for anchored/free fits; RSE, relative standard error; *R*(*T*_i_), temperature-dependent natural remanent magnetization (NRM)–to–thermal remanent magnetization (TRM) ratio used in the Triaxe method; *B*_ANC_, paleointensity estimate; *B*_LAB_, laboratory field.

Paleointensity experiments targeted sites with well-defined characteristic remanent magnetization (ChRM) directions (Fig. 2A). Bourinot samples were analyzed using thermal Thellier experiments as their weak magnetization made them unsuitable for reliable Shaw or continuous-method determinations. Itabaiana samples were investigated using complementary approaches including thermal and microwave Thellier, modified Shaw, and continuous Triaxe/Wilson methods. Representative Arai and pseudo-Arai diagrams are shown in Fig. 3 and fig. S5; full specimen-level results are reported in tables S3 to S5, with anisotropy tensors in table S6. All Thellier-type experiments were screened using SCOPE (Supplementary Note 1), and the full set of selection criteria applied across methods is summarized in table S11. Full method descriptions are provided in Supplementary Note 5.

For both localities, only sites with reliable paleomagnetic directions consistent with previous studies were retained for paleointensity analysis (Fig. 2A). At Bourinot, sites BV5, BV7, and BV8 lie near the original directional sites and match the established paleomagnetic population (*23*). The remaining sampled sites were excluded because they did not reproduce this directional population: Some specimens showed noisy, nonideal demagnetization behavior, and others, although individually more coherent, did not yield mutually consistent site-level directions. At Itabaiana, three sites from the main swarm (IT2, IT7, and IT8) and one nearby reversed-polarity site (IT1) were retained (*24*). Several N-S trending dykes with distinct directions were excluded a priori because they may represent a separate intrusive phase.

Accepted results from both localities produce well-clustered within-site means (Fig. 2B and Table 1). At Bourinot, accepted Arai slopes are linear through the fitted temperature range and coincide with origin-trending ChRM directions (Fig. 3, A to C). These experiments yield consistently ultraweak values, including estimates as low as 0.4 μT, even when stronger laboratory fields were applied, although stronger fields tended to accentuate zigzagging associated with pTRM (partial thermal remanent magnetization) tails (*30*, *31*) (Fig. 3C). Additional Bourinot Arai examples are provided in Supplementary Note 2.4 and fig. S5.

**Table 1.** Summary site-mean directional and paleointensity results for Bourinot and Itabaiana. Dec, declination; Inc, inclination; *N*_Dir_, number of specimens used for the site-mean direction; *k* and α95, Fisher statistics; *N*_INT_, number of accepted specimen-level paleointensity estimates contributing to the site mean; *B*_ANC_, site-mean paleointensity; σ_B_, SD of *B*_ANC_; VDM, virtual dipole moment; σ_VDM_, SD of VDM; *Q*_PI_, paleointensity quality index. Bourinot site means are based on thermal Thellier results only, whereas Itabaiana site means incorporate multiple methods.

Site	Age	Dec	Inc	*N*_Dir_	*k*	α95	*N*_INT_	*B*_ANC_	σ_B_	VDM	σ_VDM_	*Q*_PI_
	(Ma)	(°)	(°)			(°)		(μT)		(×10^22^ A·m^2^)		
BV5	505 ± 3	289.5	16.8	5	26.6	15.1	7	0.6	0.2	0.1	0.0	5
BV7	505 ± 3	286.4	35.0	6	90.8	7.1	5	1.7	0.5	0.4	0.1	6
BV8	505 ± 3	281.7	31.1	5	11.5	23.6	3	0.9	0.4	0.2	0.1	5
IT1	525 ± 5	41.7	52.2	5	120.1	7.0	11	8.0	2.4	1.5	0.5	9
IT2	525 ± 5	169.0	−68.9	7	150.6	4.9	20	12.2	2.8	1.9	0.4	10
IT7	525 ± 5	173.3	−65.0	6	967.1	2.2	18	11.4	2.5	1.8	0.4	9
IT8	525 ± 5	185.8	−58.1	5	47.0	11.3	11	12.3	1.9	2.1	0.3	9

At Itabaiana, five different methods were applied and the resulting paleointensities show good agreement between methods and lithologies (Fig. 2B and Table 1). Thermal Thellier estimates tend to be slightly weaker within the main site cluster, and the near opposite-polarity site IT1 is consistently weaker than the other dyke sites. Some continuous Triaxe results showed increasing intensity across the ChRM window, consistent with the documented cooling-rate dependence of single domain magnetite above ~400°C (*32*, *33*); in those cases, only lower-temperature windows were used, leading to rejection of two specimens. Shaw results obtained from baked host rocks at IT1 and IT2 are broadly consistent with the dyke results, although two required large alteration corrections and gave slightly higher estimates (Figs. 2B and 3, D to H).

Site-mean paleointensity quality is high by published standards, with quality of paleointensity index (*Q*_PI_) values ranging from 5 to 10 (Table 1 and table S7) (*34*). The new site means correspond to weak virtual dipole moments: 1.5 × 10^22^ to 2.1 × 10^22^ A·m^2^ for Itabaiana and 0.2 × 10^22^ to 0.5 × 10^22^ A·m^2^ for Bourinot (Table 1). These values extend the record of suppressed field strength into the early-middle Cambrian, beyond the onset of the Cambrian radiation.

### Reassessed field-strength synthesis across ~590 to 505 Ma

To place the new whole-rock estimates in a consistent interval-scale synthesis, previously published single-crystal paleointensity (SCP) datasets from Glen Mountains, Chatham-Grenville, and Sept-Îles were reassessed using the same screening logic. Direct comparison between new whole-rock estimates and published SCP values is otherwise confounded by inconsistent treatment of anisotropy, alteration control, Arai-slope linearity, and fit-selection permissiveness. Supplementary Note 3 documents the full specimen-level reassessment and the main sources of uncertainty across these studies.

At Glen Mountains [~532 Ma; (*4*)], application of SCOPE yields 10 accepted specimens and lowers the study mean relative to the originally published value, bringing it into closer agreement with the weak-field range defined by the Cambrian whole-rock data. At Chatham-Grenville [~544 Ma; (*3*)], only one specimen satisfies the common screening approach, preventing derivation of a robust site mean: unresolved anisotropy, low-temperature alteration, and likely cooling-rate effects further limit confidence in the published SCP values as quantitative field estimates. At Sept-Îles [~565 Ma; (*9*)], the revised distribution shifts upward because the previously emphasized ultralow mode is preferentially rejected under consistent filtering, raising the study mean by ~50% relative to the original presentation.

**Fig. 4.**
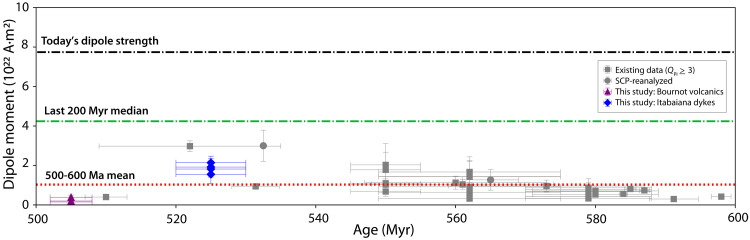
Cambrian dipole moment synthesis and reanalyzed single-crystal results. Dipole moment data [taken mainly from www.pintdb.org; (*37*)] for 500 to 600 Ma, with *Q*_PI_ ≥ 3, reported and plotted with SD. The red dotted line is the mean field for the plotted interval (600 to 500 Ma) including SCP-reanalyzed data. The green dash-dotted line is the long-term average for the last 200 million years (Myr) (*38*). Dipole moment for Bono plotted with assumed cooling rate correction but without “unknown” anisotropy corrections. The reanalyzed SCP estimates remain identifiable from their ages and distinct marker style, whereas their dataset-specific treatment is documented in the Supplementary Materials and associated tables.

Together, the new Cambrian whole-rock data and the uniformly reassessed published estimates define an interval-scale picture across ~590 to 505 Ma in which low to ultralow dipole moment persists for tens of millions of years beyond the Ediacaran-Cambrian transition (Fig. 4). The revised SCP distributions overlap more strongly with the whole-rock record than previously, and the combined dataset shows no evidence for a sharp and sustained strengthening at ~550 Ma.

## DISCUSSION

### Low dipole moments persisted into the Cambrian

The new Bourinot and Itabaiana paleointensity results provide two well-constrained additions to the early-middle Cambrian record from localities formed on different plates [Gondwana and Avalonia; e.g., (*39*)]. In all cases, the technical quality of the data is acceptable, and the results are likely free from substantial bias: Accepted fits are linear, origin-trending, and pass alteration checks on all temperatures below and across the fitted intervals. At Itabaiana, confidence is particularly high because differing experimental treatments and contrasting lithologies, including baked host rocks (Fig. 2 and tables S4 and S6), show internally coherent behavior. All results converge on, indicating that the paleomagnetic field was very weak.

Our interval-scale analysis shows that inferences of geomagnetic evolution may be sensitive to how paleointensity results are filtered. Here, SCOPE provides a consistent basis for comparison across new and published estimates. Applying the common SCOPE set of criteria changes distributions such that SCP estimates shift toward values comparable to the Cambrian whole-rock record and the entire dataset does not require a sharp and sustained rise in dipole moment at ~550 Ma.

The reassessment (Supplementary Note 3) suggests that published SCP criteria may be overly permissive and less effective at excluding weakly supported or curved fits. In addition, the datasets also carry recurring complication factors that are not fully captured by specimen-level screening alone. These include incomplete treatment of TRM anisotropy, limited low-temperature alteration control, possible cooling-rate effects in slowly cooled intrusive material, and, in some cases, uncertainty over whether analyzed remanences are primary TRMs. These factors do not invalidate SCP measurements, but they do affect how much weight can be placed on individual estimates in a long timescale synthesis.

When the new whole-rock results are combined with the uniformly reassessed SCP datasets, the record across ~590 to 505 Ma suggests that dipole moment remained persistently low rather than undergoing a rapid post-Ediacaran recovery (Fig. 4).

### A weak and unstable Ediacaran-Cambrian geodynamo

The persistently weak paleomagnetic field intensity recorded across the 500- to 600-Ma interval is consistent with growing evidence for reversal hyperactivity spanning much of the latest Ediacaran and early-middle Cambrian (*40*–*42*). Dual polarities are present directly in the Itabaiana and Bourinot records (*23*, *24*), and independent directional studies point to unusually frequent reversals across the broader transition interval (*40*–*42*). The combined picture is therefore not simply one of low field strength but of a geodynamo that remained weak and unstable for tens of millions of years. The paleointensity data presented here do not by themselves distinguish between a persistently weak average field and one that was highly variable with repeated weak intervals associated with reversals, but either scenario implies a field state markedly different from the strong dipole regime characteristic of much of later Earth history.

### Implications for ICN and magnetospheric shielding

The results presented here do not rule out ICN near ~550 Ma, but neither do they provide observational support for a sharp and sustained field strengthening at that time. Some thermal evolution models permit a wide range of possible ICN ages and do not require a pronounced intensity jump at nucleation (*18*, *20*, *21*). The paleointensity record across ~600 to 500 Ma therefore constrains not whether ICN occurred but how clearly any change in geodynamo behavior was expressed in field strength.

If geomagnetic field strength remained weak for tens of millions of years after the onset of the Cambrian radiation, magnetospheric shielding also remained suppressed through a key interval of early animal evolution. This does not establish a direct causal role for geomagnetic change in biological or environmental transitions because nutrient supply, redox evolution, and evolutionary innovation all remain plausible drivers (*10*–*15*). It does, however, appear incompatible with hypotheses that require rapid restoration of strong shielding immediately after the Ediacaran. Together, the new Bourinot and Itabaiana results and the reassessed SCP synthesis instead support continued geomagnetic weakness and an unstable, reversal-prone field into the Cambrian. Additional high-quality paleointensity and directional data from the 600- to 500-Ma interval will be needed to test more rigorously how hypotheses involving ICN relate to field behavior and surface environmental change during this interval of Earth history.

## MATERIALS AND METHODS

### Rock magnetic and petrographic methods

Rock magnetic experiments were performed on representative specimens from each site to characterize remanence carriers, thermomagnetic behavior, and domain-state indicators. Hysteresis, high-field saturation magnetization versus temperature, and high-temperature susceptibility measurements were used to assess thermal stability and mineralogical behavior. Thin sections from selected Bourinot and Itabaiana sites were examined using backscatter electron imaging and EDX to characterize Fe-Ti oxide textures and associated opaque phases. Additional microscopy and EDX information is provided in Supplementary Note 2.

### Paleointensity experiments

Paleointensity experiments targeted samples with well-defined ChRMs over appropriate temperature intervals. Methods were chosen according to rock-magnetic behavior and signal strength. Bourinot samples were investigated using thermal Thellier experiments with the IZZI+ protocol, whereas Itabaiana samples were analyzed using complementary methods including thermal and microwave Thellier, modified Shaw, and continuous Triaxe/Wilson approaches. This combination was intended to provide independent checks on alteration, domain-state effects, and anisotropy-related bias. Full method-specific details are provided in Supplementary Note 5.

### SCOPE and reassessment of published estimates

All Thellier-type experiments were screened using SCOPE, a set of selection criteria derived from the paleointensity literature and tested against known-field experiments. All selection criteria applied in this study, including the SCOPE thresholds and the corresponding criteria used for the other paleointensity methods, are listed in table S11. Previously published estimates from the critical Ediacaran-Cambrian interval were reassessed under the same approach to allow internally consistent comparison with the new whole-rock results. The derivation and validation of SCOPE are given in Supplementary Note 1, the specimen-level reassessment is detailed in Supplementary Note 3, and the treatment of the previously published Chatham-Grenville whole-rock estimate is documented in Supplementary Note 4.

## Supplementary Materials

### The PDF file includes:

Supplementary Notes 1 to 5 Figs. S1 to S7 Legends for tables S1 to S11 References
