# Supplementary material for: Earth’s magnetic field remained weak for 40 million years after the Cambrian radiation
Source: Sci Adv. 2026 Jul 23;12(30):eaeg2325. doi: 10.1126/sciadv.aeg2325 (PMC13394383; doi:10.1126/sciadv.aeg2325)
Supplement: Supplementary file 1 — Supplementary Notes 1 to 5 Figs. S1 to S7 Legends for tables S1 to S11 References [file sciadv.aeg2325_sm.pdf]

Science Advances
AAAS

Supplementary Materials for

Earth's magnetic field remained weak for 40 million years after the Cambrian radiation

Simon J. Lloyd *et al.*

Corresponding author: Simon J. Lloyd, sjlloyd@liverpool.ac.uk

Sci. Adv. **12**, eaeg2325 (2026)
DOI: 10.1126/sciadv.aeg2325

This PDF file includes:

Supplementary Notes 1 to 5
Figs. S1 to S7
Legends for tables S1 to S11
References

Other Supplementary Material for this manuscript includes the following:

Tables S1 to S11

Supplementary Note 1. Definition and performance of SCOPE

Selection Criteria Optimised for Palaeointensity Experiments (SCOPE) was defined to provide a transparent, literature-grounded, internally tested, and not overly restrictive basis for screening Thellier-type palaeointensity results in this study and for reassessing previously published single-crystal palaeointensity (SCP) datasets used in the comparative synthesis. The aim was to establish a common screening approach that was neither ad hoc nor strongly study-specific, but instead based on the range of criteria already used in the palaeointensity literature. SCOPE is applied here to the new whole-rock Thellier-type results and to the reassessment of published SCP datasets.

The criteria were compiled from 27 published palaeointensity selection-criteria sets from the last ~15 years for rocks aged ~300–3000 Ma and yielding results with quality of palaeointensity index (Q_{PI}) ≥ 3. These include the published SCP studies that are reassessed here. For each of the seven most commonly reported parameters, SCOPE adopts the relaxed outer-quartile threshold from the published literature, thereby defining a moderately permissive but literature-grounded filter. The individual parameter distributions are shown in Supplementary Figure S1A. The literature compilation used to define SCOPE, and the resulting outer-quartile thresholds are provided in Supplementary Table S1. All selection criteria applied in this study, including the SCOPE values and the corresponding criteria for the other palaeointensity methods, are listed in Supplementary Table S11.

The performance of SCOPE was tested using historical datasets on rocks with known thermal remanent magnetisation (TRM;25–27); this consisted of 489 specimens generating ~44,000 possible Arai fits with n ≥ 3. A basic physical screen was first applied to remove clearly spurious fits (accepting fits with $b < 0$, $f \geq 0.15$, $g > 0$), yielding a working dataset of ~34,000 fits. Accuracy was assessed using the mean (m) and scatter (s) of ln (B_{anc}/B_{exp}), and discrimination was measured using $S = E_a \times E_i$ (25), where accurate fits lie within ±10% of the known field. When applied to the historical dataset, SCOPE produces high accuracy m, low s, and high discrimination scores comparable to stricter whole-rock criteria, indicating that it effectively accepts accurate fits while rejecting inaccurate ones (Supplementary Figure S1B–E; Supplementary Table S2).

SCOPE occupies a favourable region of parameter space. Relative to alternative published criteria sets, it achieves strong rejection of inaccurate fits while retaining a substantial number of accepted fits. The result is a balanced compromise between bias, scatter, discrimination, and data yield. On that basis, SCOPE provides a consistent and justifiable filter for both the new whole-rock data presented here and the comparative reassessment of previously published SCP datasets.

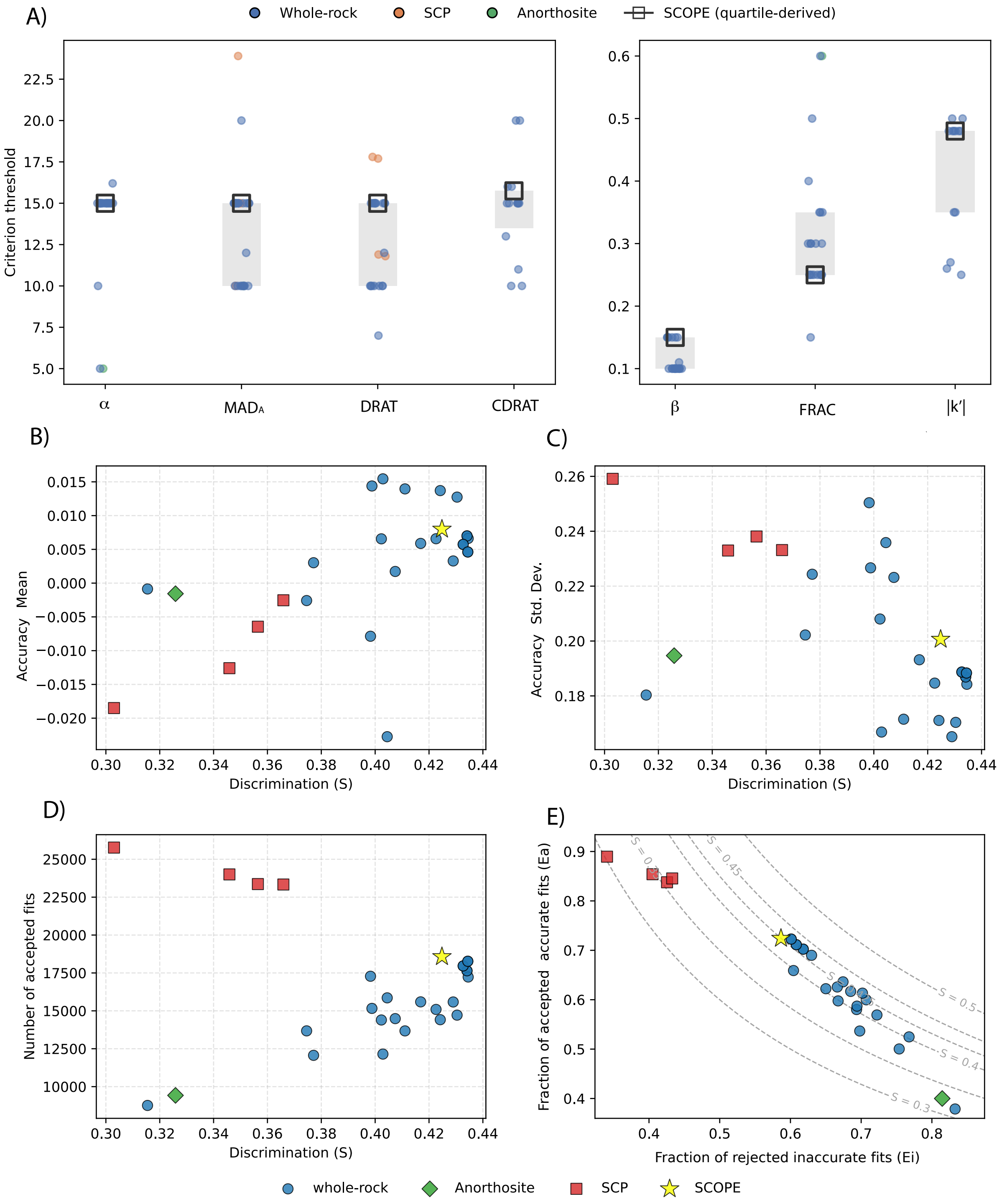

Supplementary Figure S1. Definition and performance of SCOPE selection criteria. (A) Compilation of 27 published palaeointensity selection-criteria sets spanning the last ~12 years for rocks aged ~300–3000 Ma ($Q_{PI} \geq 3$; 34). For the seven most commonly reported parameters, literature values are shown by study type (whole-rock, single-crystal (SCP), anorthosite), with shaded boxes indicating the interquartile range. SCOPE thresholds (open squares) are defined by adopting the outer quartile

corresponding to the most relaxed commonly used value for each parameter. (B) Mean accuracy and (C) accuracy scatter as a function of discrimination (*S*; 25), for each criteria set; higher *S* corresponds to improved discrimination between accurate and inaccurate fits. (D) Number of accepted fits as a function of *S*, illustrating the trade-off between discrimination and data yield. (E) Relationship between the fraction of rejected inaccurate fits (E_i) and the fraction of accepted accurate fits (E_a), with dashed curves showing contours of constant $S = E_a \times E_i$. The SCOPE criteria (star) occupy a region of high discrimination while retaining a substantial number of accepted fits.

Supplementary Note 2. Additional support for the rock magnetic, microscopy, and remanence-behaviour interpretation

2.1 Detailed rock magnetic and petrographic methods

Rock magnetic experiments were performed on representative samples from each site to characterise the magnetic mineralogy and thermomagnetic properties. Hysteresis loops, backfield coercivity, and high-field thermomagnetic curves of saturation magnetisation (*Ms*–*T*) were obtained using a Magnetic Measurements Ltd Variable Field Translation Balance. High-temperature susceptibility (*k*–*T*) experiments were carried out to either 600 °C, 700 °C, or as cyclic experiments of increasing temperature using an MFK1-FA Kappabridge susceptometer with a CS-3 furnace (AGICO). Thin sections were produced from sites BV5, BV7, BV8, IT1, IT2, and IT7. These were analysed on a high-resolution, variable-pressure Zeiss Gemini SEM 450 (University of Liverpool SEM Shared Research Facility) with backscatter electron imaging (BSE) and energy-dispersive X-ray spectroscopy (EDX) used for mineral identification and semi-quantitative elemental analysis.

2.2 Detailed thermomagnetic observations

Thermomagnetic curves derived from Bourinot samples indicate stable, largely reversible behaviour (Supplementary Figure S2A). *k*–*T* curves are almost closed on heating and cooling, with only mild separation appearing above ~550–600 °C; *Ms*–*T* curves are similarly reversible. Two Curie temperatures are typically observed at ~570 °C and ~630 °C, corresponding to low-Ti titanomagnetite and titanohematite phases. Hopkinson-like peaks are modest, and magnetisation is weak, producing noisy *k*–*T* curves but supporting thermal stability. Slightly negative *Ms*–*T* values likely result from the weakly magnetised specimens being affected by background diamagnetic material, including the quartz wool, and from instrument limits.

Across Itabaiana sites, Curie temperatures cluster around 580 °C (Supplementary Figure S2B), consistent with Trindade et al. (24). *k*–*T* curves have pronounced Hopkinson-like peaks in some cases and are reversible up to at least 550 °C, exhibiting varying degrees of alteration at higher

temperatures. Paired *Ms–T* traces for representative specimens are likewise stable to the same threshold, with irreversible change confined to the highest temperature sections. On the Day plot (Supplementary Figure S2C), Itabaiana sites fall in the upper-left region, consistent with fine primary remanence carriers (e.g., 28, 29). Together, these observations indicate near-stoichiometric magnetite-dominated remanence with single-domain to single-vortex behaviour and negligible low-temperature alteration during heating.

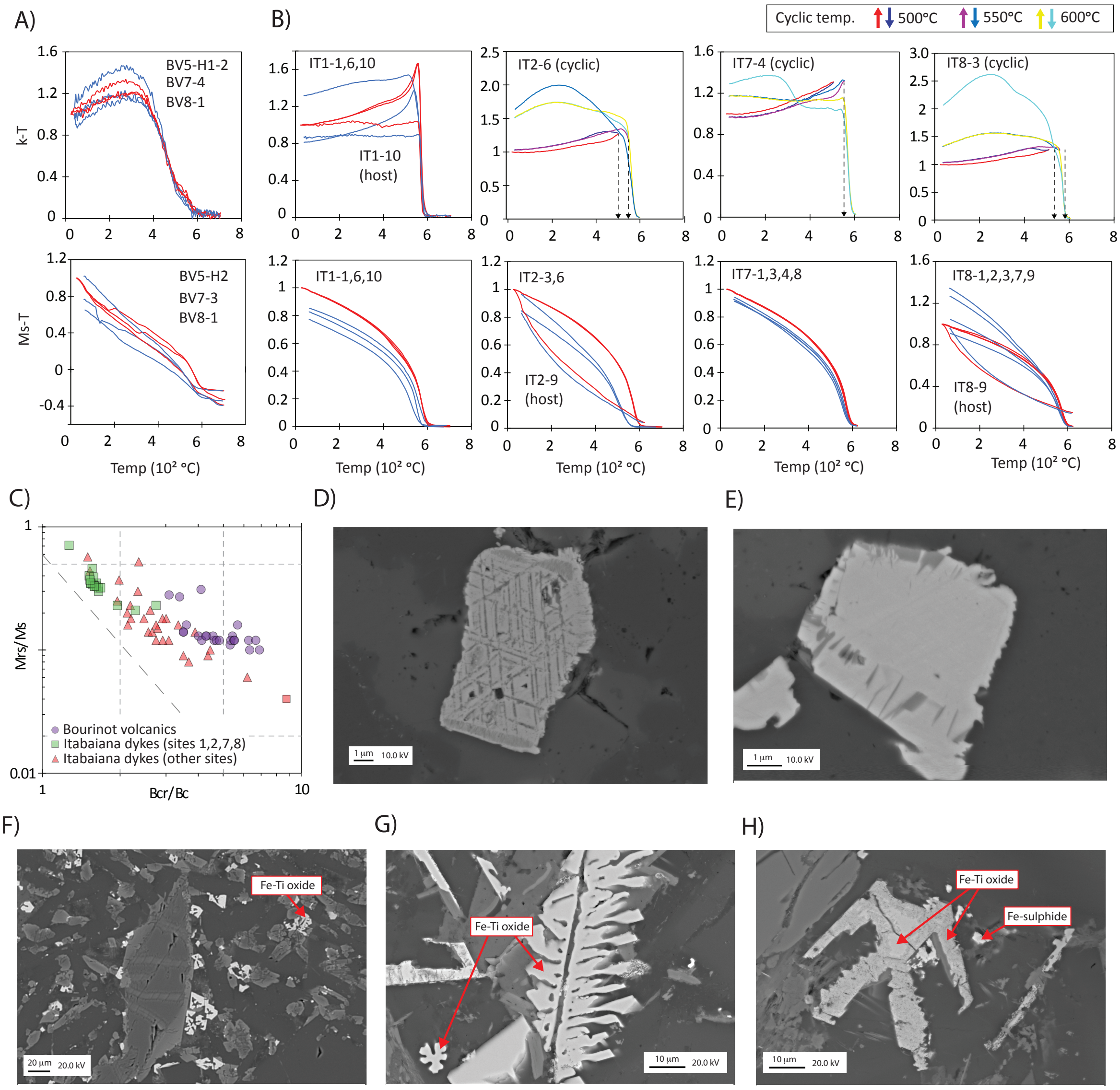

Supplementary Figure S2. Results from rock magnetic and scanning electron microscopy. (A) *k–T*/*Ms–T* normalised curves for Bourinot are reversible. Slightly negative *Ms–T* values likely result from the weakly magnetised specimens being affected by background diamagnetic material including the quartz wool, and from instrument limits. (B) *k–T*/*Ms–T* curves for Itabaiana; some with cyclic heating (as indicated). Curves display pronounced Hopkinson peaks and are reversible to ≥ 550–600 °C. (C) Day

plot (*Mrs/Ms* vs. *Bcr/Bc*). Itabaiana dyke specimens (separate sites IT1, IT2, IT7, IT8) cluster in the SD–PSD field, whereas Bourinot volcanics have lower Mrs/Ms and higher *Bcr/Bc* values. d-h) BSE images. (D) BV5 Fe-Ti oxide with a dense trellis of planar lamellae in a Fe-richer host. (E) BV7 Fe-Ti oxide with sharp margins and straight, cross-cutting lamellae in a magnetite/titanomagnetite host. (F) IT1 plate-like opaques with sharp, straight rim and uniform interior (Supplementary Figure S4, spectrum 22). (G) IT2 skeletal/dendritic opaque with crisp branching and clean boundaries (Supplementary Figure S4, spectrum 10). (H) IT7 skeletal/lath Fe–Ti oxide plate cutting the groundmass at high angle, showing straight outer rims and subdued internal contrast. In panels D–H, the dark areas are the silicate background matrix.

2.3 Additional microscopy observations

Opaque grains in the Bourinot volcanics are mostly equant to sub-equant with smooth, sharp rims. Several grains contain planar lamellae intersecting at ~60° and are Ti-enriched relative to their low-Ti Fe-oxide hosts. The limited development of these lamellae, together with the absence of pervasive fine trellis and of late mottled or pseudomorphic textures, indicates mild oxidation during cooling. Comparison with a published example of oxidation-exsolution texture in Fe–Ti oxide solid solutions (43; Supplementary Figure S3) shows that the Bourinot grains are more comparable to early lamellar/trellis oxidation textures than to later pseudomorphic replacement, although the Bourinot lamellae are less developed than in the published example.

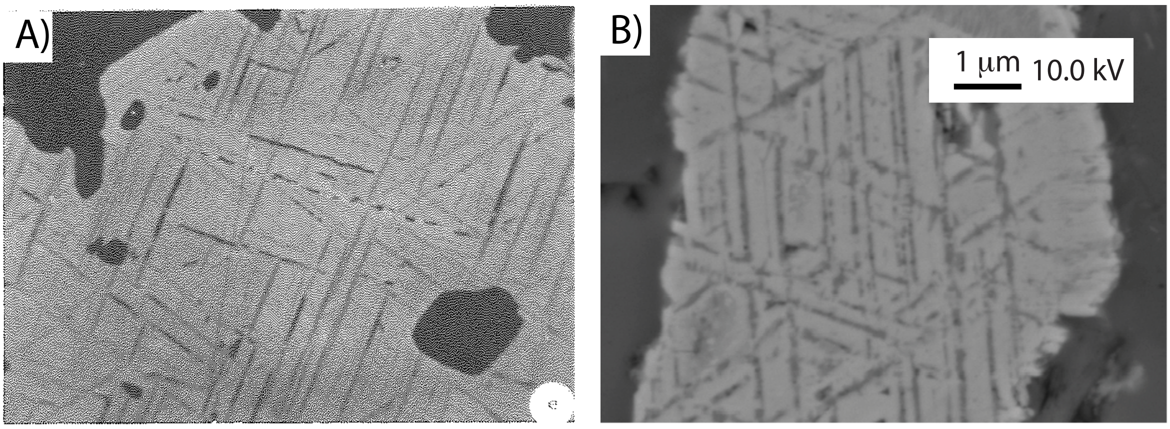

Supplementary Figure S3. Comparison of Bourinot Fe–Ti oxide texture with a published example of oxidation-exsolution in Fe–Ti oxide solid solutions. (A) Reproduced example from Haggerty (43), Figure 6E, showing early lamellar/trellis oxidation texture; reproduced with permission from the Mineralogical Society of America. (B) Reproduced Bourinot BSE image from Supplementary Figure S2D, shown again here for direct comparison. The reproduced Haggerty panel is shown at its original scale (0.15 mm field width), which is approximately twice the width of panel b. The comparison is intended to illustrate similarity in oxide-texture style, not equivalence of whole-rock lithology.

Itabaiana back-scattered electron images (Supplementary Figure S2F–H) show opaque phases dominated by Fe–Ti oxides with sharp, straight external rims and uniform interiors at the scale imaged. Three habits are observed: slender laths or veinlets cutting the groundmass at high angles; skeletal-dendritic plates with short lateral branches; and crisp, irregular branching networks. Internal contrast is subdued; there is no pervasive mottling or pseudomorphic replacement, and no fine trellis of intersecting lamellae indicative of advanced oxy-exsolution. Point EDX confirms Fe–Ti oxides of moderate to Ti-rich composition (Supplementary Figure S4A–C, F–G). Some brighter, rounded nodules occur adjacent to the oxides but remain texturally discrete: they neither rim nor replace the Fe–Ti phases and do not form interconnected networks. EDX identifies these as Fe-sulphides with minor Ni (Supplementary Figure S4D, E, H). Overall, the oxide textures are crisp and uniform, with no late low-temperature replacement fabrics observed at this scale. The sulphides remain texturally distinct from the Fe–Ti oxides, supporting the inference that the thermomagnetic behaviour is associated with the oxide assemblage rather than with sulphides.

Although some of the visible Fe–Ti oxide grains are large, the clean Arai behaviour and hysteresis properties suggest that the dominant remanence is likely carried by a finer magnetic fraction, or by magnetic substructures not resolved at the scale of the present observations.

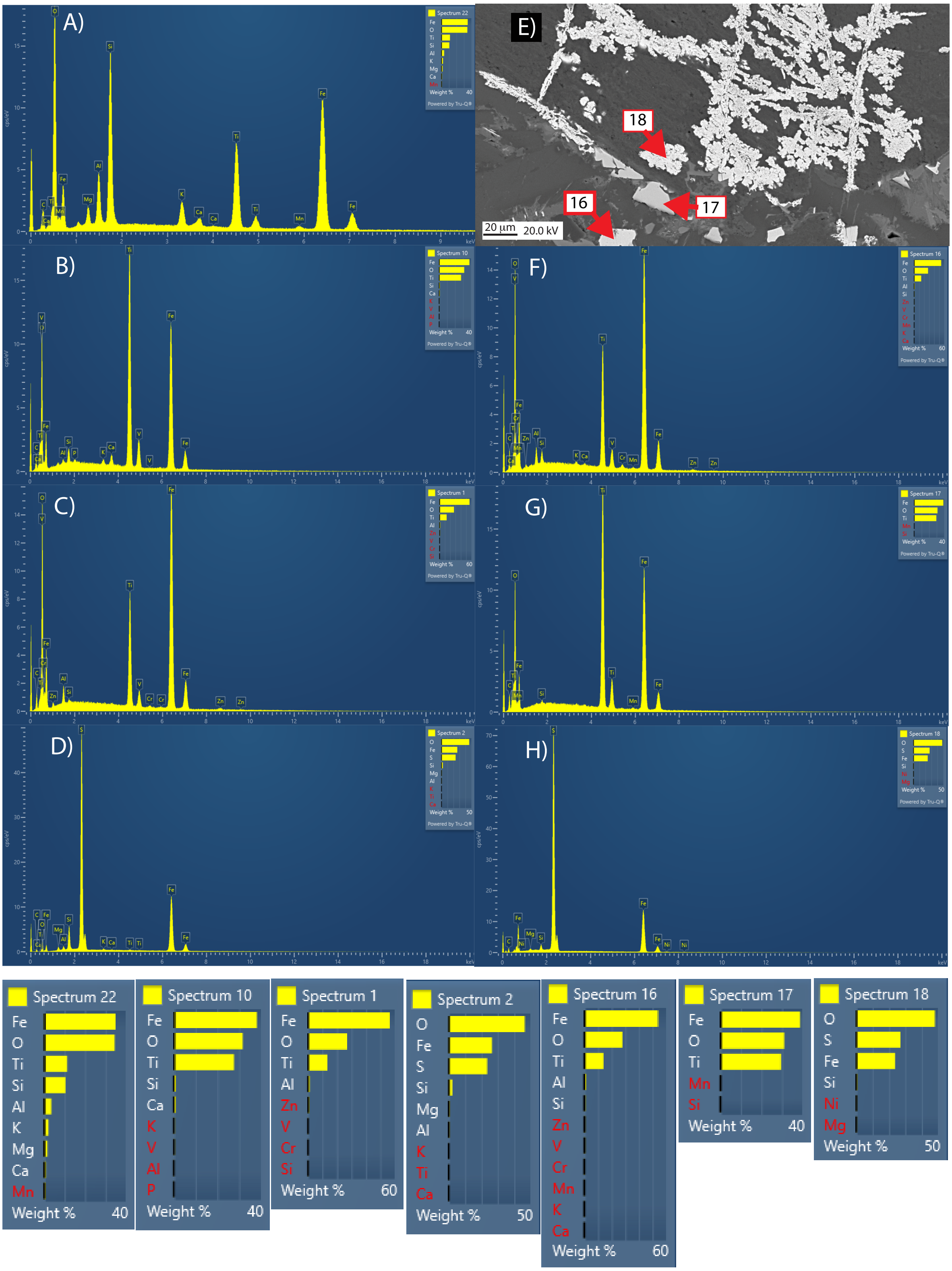

Supplementary Figure S4. SEM–EDX confirmation of phase identifications associated with the Itabaiana BSE panels shown in Supplementary Figure S2F–H. (A) IT1 grain, Fe–Ti oxide (Spectrum 22; corresponding to Supplementary Figure S2f). (B) IT2 grain, Fe–Ti oxide (Spectrum 10; corresponding to Supplementary Figure S2G). (C, D) IT7 grains from Supplementary Figure S2H, showing a Fe–Ti oxide in the central grain (Spectrum 1) and an adjacent Fe-sulphide (Spectrum 2). (E) IT2 BSE image showing

the locations of Spectra 16–18. (F, G) Fe–Ti oxides from panel (E) (Spectra 16 and 17). (H) Fe-sulphide from panel (E) (Spectrum 18). Panel (E) shows that sulphides occur adjacent to, but do not rim or replace, the Fe–Ti oxides. Their spatial separation and distinct chemistries support the interpretation that the thermomagnetic behaviour described in the main text and Supplementary Note 2 is associated with the Fe–Ti oxide assemblage rather than with sulphides. The same spectra side bars are shown enlarged at the bottom of the figure.

2.4 Additional support from Bourinot Arai behaviour

Additional Bourinot Arai diagrams are shown in Supplementary Figure S5, with intermediate temperature steps labelled. The accepted slopes remain approximately linear across the magnetite and higher-temperature oxide ranges discussed in the text. Although this behaviour does not by itself demonstrate a pure TRM origin, it suggests that the accepted fits are not obviously dominated by a separate low-temperature chemical component. As a precaution, Q_{TRM} was set to zero for the Bourinot sites in the Q_{PI} assessment (Supplementary Table S7).

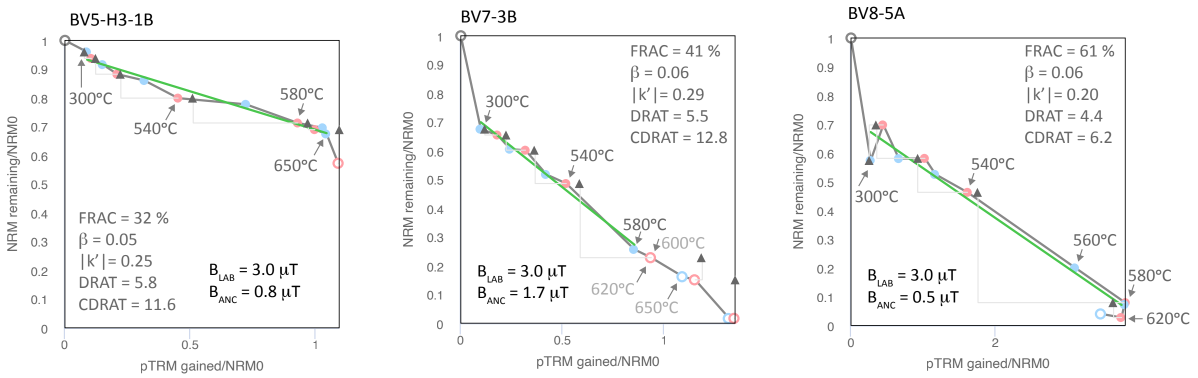

Supplementary Figure S5. Additional Bourinot Arai diagrams. These show accepted thermal Thellier behaviour with intermediate temperature steps labelled. These examples illustrate that accepted slopes remain approximately linear across the magnetite and higher-temperature oxide ranges discussed in the text.

Supplementary Note 3. Reanalysis of published single-crystal palaeointensity datasets

This note documents the uniform reanalysis applied to previously published single-crystal palaeointensity (SCP) datasets (3, 4, 9) incorporated into the comparative synthesis used in this study. The purpose is to place published specimen-level results on a more consistent footing by applying the same performance-tested selection criteria (SCOPE) across studies, while also examining additional methodological and reporting issues that bear directly on quantitative comparison. These subsequent analyses are required to make the synthesis robust, and to clarify how recurring sources of uncertainty affect interpretation of the published SCP datasets.

3.1 Reproducibility of the SCP reanalysis workflow

The reanalysis of previously published SCP datasets was carried out using paleointensity.org, which reads the original study files downloaded directly from the MagIC community database. No reformatting of those files was required prior to screening. The specimen-level supplementary tables presented here report the reanalysed results used in the comparative synthesis; the corresponding input files are the original publicly available MagIC downloads associated with the published studies and can be retrieved directly for reproduction of the workflow in paleointensity.org. An exception is Zhou et al. (3), for which the format of the MagIC upload prevented this workflow from being followed; that reanalysis was instead carried out using the authors' Figshare dataset.

3.2 General factors affecting SCP interpretation

Several recurring issues affect quantitative interpretation across the SCP datasets considered here. First, when evaluated against the same large historic dataset of known-field palaeointensity experiments used to assess SCOPE (Supplementary Note 1), published SCP criteria yield systematically lower discrimination scores than both whole-rock criteria and SCOPE. Low discrimination scores are accompanied by poorer accuracy performance, expressed as both greater scatter and weaker rejection of inaccurate fits (Supplementary Figure S1E; Supplementary Table S2).

In particular, SCP criteria cluster at low E_{i}, indicating weak rejection of inaccurate fits, while simultaneously retaining fits with large dispersion (s). This behaviour contrasts with most whole-rock criteria, which achieve higher discrimination by more effectively excluding inaccurate fits, even where this modestly reduces E_{a}. The single whole-rock outlier (Ref 18 in Supplementary Table S2) reflects overly restrictive DRAT (maximum absolute pTRM-check difference normalised by the length of the best-fit line) and FRAC (fraction of remanence used for the best-fit segment) thresholds that suppress E_{a} without a commensurate gain in E_{i}, highlighting the trade-off inherent in excessively strict filtering.

In practice, permissive SCP criteria tend to retain a larger proportion of inaccurate or weakly constrained fits than criteria sets that achieve better balance between data yield and screening performance. On this basis, SCOPE provides a more consistent and defensible filter for cross-study comparison.

Second, TRM anisotropy remains a material source of uncertainty. SCP estimates are especially vulnerable to anisotropy bias in anorthositic material, where crystallographically controlled magnetite inclusions can produce strong directional dependence of TRM acquisition (44–47).

Across the three SCP studies, specimen-level ATRM tensors are not reported: corrections are partial or undocumented (9), applied only to a limited subset (3), or absent despite large directional offsets (4). Where this is the case, anisotropy bias cannot be excluded as a contributor to the reported field estimates.

Third, accepted T_{max} values in all three studies cluster around ~400 °C, implying remanence carried predominantly by ~TM30 titanomagnetite. This composition has recently been shown to be problematic for determining absolute palaeointensity (48), and therefore introduces an additional common source of uncertainty across the datasets.

Fourth, use of R^2 is not sufficient to assess the suitability of accepted Arai segments, because high correlation can coexist with substantial curvature. The curvature parameter |k'| (49) is the more relevant measure of the linearity required for Thellier-type analysis. Finally, low-temperature alteration may remain undetected where partial TRM (pTRM) checks are sparse or absent below ~400 °C (3, 4), and recomputation of DRAT values following the Standard Paleointensity Definitions SPD v1.2.0 (25) shows that reported values can underestimate alteration where early pTRM checks are omitted from the calculation (Supplementary Table S8; Supplementary Figure S6).

Taken together, these issues do not preclude SCP measurements from contributing useful information, but they do require cautious and internally consistent treatment when SCP datasets are compared with each other and with whole-rock results in a synthesis spanning the Ediacaran–Cambrian interval.

3.3 Glen Mountains anorthosite (plagioclase; ~532 Ma)

Application of SCOPE to the Glen Mountains plagioclase dataset yields ten accepted specimens. Several specimens are rejected owing to non-linear Arai behaviour, and one specimen (gc2-1-17) is excluded because it's high γ (angle between the final pTRM and the lab field) indicates potential anisotropy bias that is not adequately constrained. Using the original authors' applied anisotropy and cooling-rate corrections, the recalculated study mean is 11.8 ± 3.1 μT, corresponding to a dipole moment of $3.0 \pm 0.8 \times 10^{22}$ Am^2 (Supplementary Table S9).

Relative to the originally reported result, the revised mean is lower and overlaps more closely with the weak-field range defined by the whole-rock record. The principal changes arise from exclusion of curved or otherwise weakly supported fits under a uniform filtering approach, rather than from any change to the original correction factors used by the authors. The Glen Mountains dataset is therefore retained in the comparative synthesis as a published SCP estimate that

remains informative, but whose quantitative contribution is best represented by the revised SCOPE-filtered mean.

3.4 Chatham–Grenville syenite (plagioclase; ~544 Ma)

Application of SCOPE to the Chatham–Grenville plagioclase dataset yields one accepted specimen with an intensity of 7.0 μT, corresponding to ~1.76×10^{22} Am^2 (Supplementary Table S8). Although this accepted value falls within the weak-field range relevant to the present synthesis, several features limit confidence in the dataset as a robust quantitative constraint on field strength. These include likely uncorrected TRM anisotropy, evidence for low-temperature alteration, and the potential for substantial uncorrected cooling-rate effects.

Reconstruction of the laboratory-field geometry indicates large anisotropy angles ($\gamma \approx 22$–55°), yet no specimen-level anisotropy measurements are reported. On equal-area projections, pTRM vectors are scattered but remain closely aligned with the characteristic remanent magnetisation (ChRM) direction (e.g., QS3-plag7; Supplementary Figure S6A), a pattern consistent with TRM anisotropy. Reducing pTRMs and failed early checks within the same specimen also indicate low-temperature alteration (Supplementary Figure S6B). In addition, reanalysis of the raw data reveals early pTRM-check failures that are not reflected in the published DRAT values, because the reported DRAT calculations omit earlier checks (e.g., QS3-Plag5; Supplementary Figure S6C, D). These features increase uncertainty in the accepted values and make it difficult to assess the magnitude of the bias from the published data alone.

Cooling-rate effects are also plausible for a syenite body of this scale. Zhou et al. (4) refer to a Curie-window cooling time of ~1 Myr, but no quantitative thermal model is provided. Given the mapped size of the intrusion (~2 km across), cooling through the relevant temperature range over ~10^4–10^5 years appears more plausible and would warrant consideration of a non-negligible cooling-rate correction; uncorrected bias can inflate field estimates for slow-cooled units (50–53). Taken together, these issues mean that the published Chatham–Grenville SCP values should be

treated cautiously and are not used here as robust quantitative constraints on field strength.

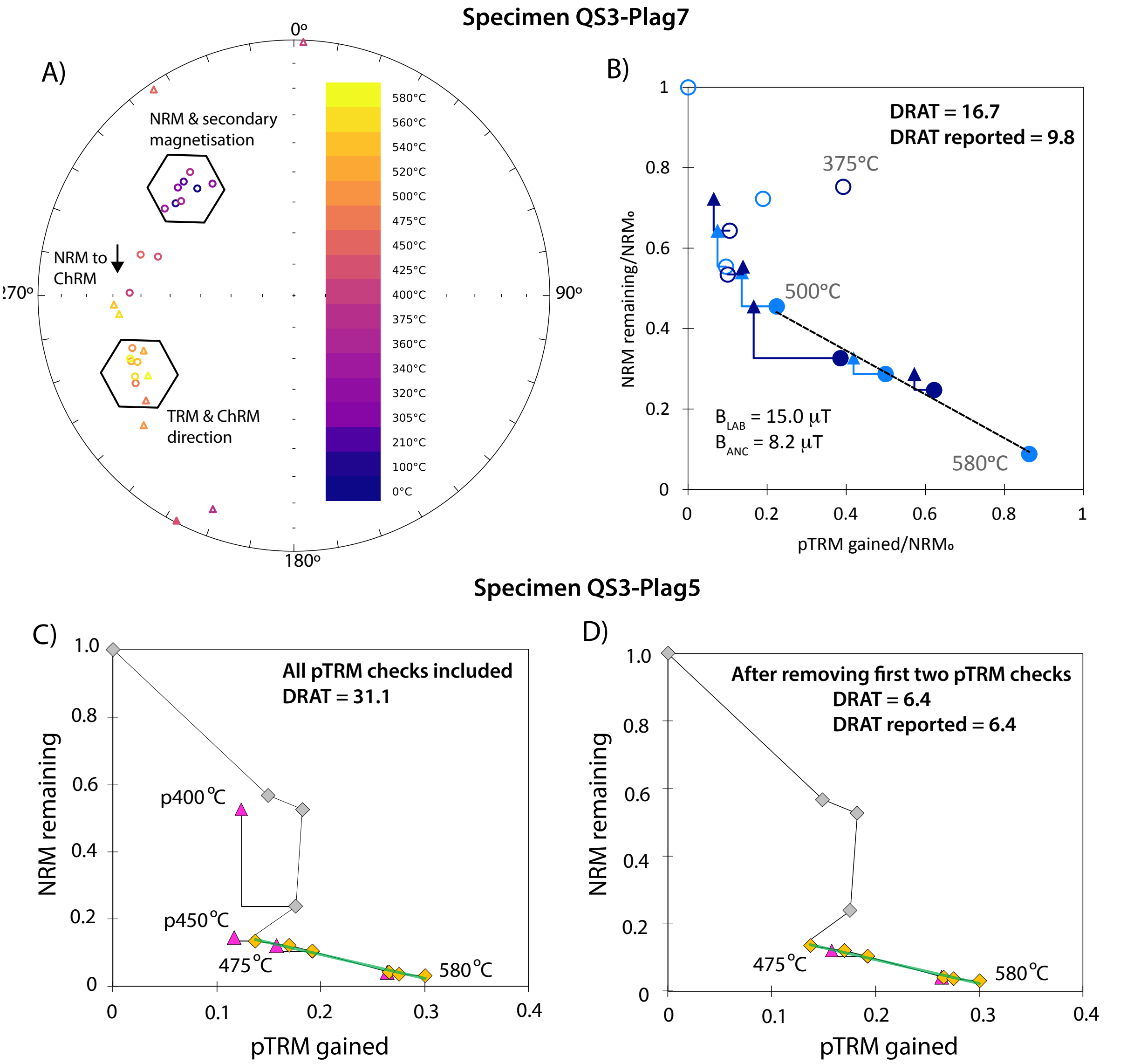

Supplementary Figure S6. Reanalysed specimens qs3-plag7. (A, B) and qs3-plag5 (C, D) from Zhou et al. (4). (A) Equal-area projection showing NRM (circles) and pTRM (triangles) directions at each temperature step; open (filled) symbols denote northern (southern) hemisphere. (B) Arai diagram for the same specimen. Open (filled) circles mark steps not used (used) in the original estimate; light blue, ZI pairs; dark blue, IZ pairs; triangles, pTRM checks; dashed line, best-fit slope. (C, D) An example of incorrect calculation of DRAT parameter (see SPD; 25). The reported value of 6.4 can only be achieved by excluding the first two pTRM checks.

3.5 Sept-Îles anorthosite (plagioclase, clinopyroxene; ~565 Ma)

Bono et al. (9) reported exceptionally weak late-Ediacaran SCP estimates and interpreted them as evidence for one of the weakest geomagnetic fields in Earth history. Application of SCOPE preferentially rejects the low-intensity mode of the bimodal dataset, while most higher-intensity results satisfy the criteria (Supplementary Table S10). As a result, the revised study mean increases by approximately 50% relative to the originally reported value. This shift does not arise from arbitrary tightening of the filter, but from differential retention of the two modes under a consistent (SCOPE) selection criteria. The reliability of the bimodal structure, rather than the original mean alone, is therefore central to interpretation of the Sept-Îles results.

Some reporting inconsistencies are also present in the published dataset. For example, certain FRAC values are not reproducible from the raw data, although these discrepancies do not materially alter the final SCOPE-filtered outcome. As in the other SCP studies, anisotropy remains an unresolved source of uncertainty: only an unspecified subset of specimens appears to have received anisotropy correction, while reported γ values span ~1–86°, with several >40°. In the absence of specimen-level ATRM tensors and complete reporting, the magnitude of anisotropy bias cannot be assessed directly. The Sept-Îles dataset is therefore retained in the comparative synthesis, but its most defensible representation is the SCOPE-filtered distribution rather than the originally emphasised ultra-low subset.

An additional uncertainty is whether the remanence analysed in the Sept-Îles feldspars represents a primary TRM. Bono and Tarduno (54) reported closely spaced crystals with opposing polarities from this material and interpreted this to indicate that exsolution of the magnetite inclusions extended to temperatures below the main unblocking range. If so, some crystals may record a thermochemical remanence rather than a pure TRM. This possibility is difficult to quantify directly from the published data, but it further supports cautious treatment of the Sept-Îles estimates, including those of Bono et al. (9), in the comparative synthesis.

3.6 Outcome for the comparative synthesis

Application of a uniform reassessment framework to the published SCP datasets produces a more self-consistent set of specimen- and study-level estimates for the Ediacaran–Cambrian interval. The revised Glen Mountains mean remains compatible with a weak-field interpretation but is lower than originally reported. The Chatham–Grenville SCP dataset is too poorly constrained to serve here as a robust quantitative field estimate. At Sept-Îles, consistent filtering preferentially removes the low-intensity mode and shifts the study mean upward, indicating that the originally emphasised ultra-weak values are not the most reliable part of the dataset.

In combination, these outcomes reduce the apparent contrast between published SCP results and the whole-rock record. The revised SCP distributions overlap more strongly with weak-field whole-rock determinations from the same broad interval and do not require a sharp and sustained rise in dipole moment at ~550 Ma. For the purposes of the present synthesis, the SCP datasets are therefore used in their SCOPE-reassessed form and interpreted with explicit caution regarding anisotropy, alteration, curvature, and cooling-rate effects.

Supplementary Note 4. Technical basis for treatment of the Chatham–Grenville whole-rock estimate in the comparative synthesis

This note documents the basis on which the previously published Chatham–Grenville whole-rock palaeointensity estimate is treated in the comparative synthesis used in this study. It considers only those issues that materially affect interpretation of the remanence age, the validity of the palaeointensity determination, or comparability with the other estimates discussed in the main text. Lloyd et al. (55) reported whole-rock palaeointensity results from a ~590 Ma Grenville dyke thermally overprinted within the aureole of the Chatham–Grenville stock and interpreted the remanence as an Early Cambrian overprint acquired during emplacement and cooling of the stock. Because this estimate lies within a key interval for the Cambrian field synthesis presented here, its treatment requires explicit documentation. Subsequent discussion, particularly Zhou et al. (4), has raised questions concerning directional coherence between thermal and alternating field (AF) demagnetisation results, possible bias from lightning overprint or pTRM-tail effects, the inferred remanence carrier and temperature range, and the age and extent of thermal resetting. These points are evaluated below only insofar as they affect treatment of the estimate in the present synthesis.

4.1 Directional coherence and origin-trending behaviour

Additional thermal demagnetisation specimens, not used in the original palaeointensity calculations but still informative for directional behaviour, were examined together with the original AF results and published site-mean directions from the Chatham–Grenville and Mont Rigaud intrusions (56). Thermal and AF populations are not strictly identical under Watson's F test (57; 58, §11.3.4), but the angular difference between their mean directions is small, both populations are tightly clustered, and both remain origin-trending. This behaviour is not consistent with substantial randomisation by lightning or with large directional distortion arising from strong unresolved pTRM-tail effects (Supplementary Figure S7).

The α-statistic, defined as the angular difference between free and anchored best-fit lines, is also low across the relevant methods: 5.3–5.6° for the three Thellier results, 8.6° ± 4.2° for Shaw (n = 9), 5.0° for microwave (n = 1), and 4.0° ± 2.0° for pseudo-Thellier (n = 5). These values indicate

that anchored and unanchored fits are similar, supporting an origin-trending high-temperature component and arguing against substantial unresolved overprint in the accepted results. Rock-magnetic observations are likewise more consistent with a narrow single-domain-dominated population than with pervasive multi-domain behaviour, further reducing the likelihood that pTRM-tail bias materially affects the reported palaeointensity values.

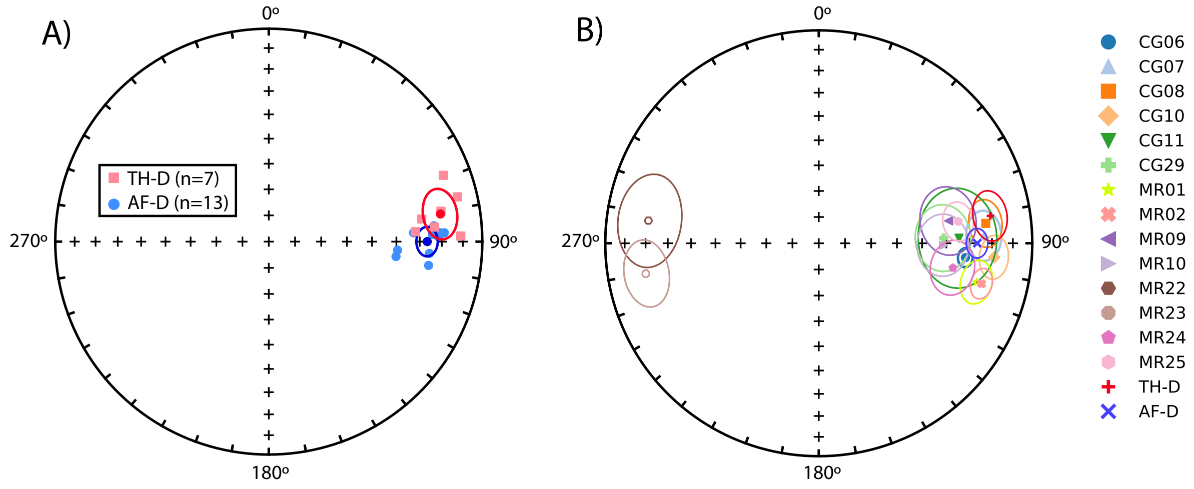

Supplementary Figure S7. Directional analysis. (A) Our specimen results (one per core) from thermal and AF demagnetisation (TH-D and AF-D) from the Chatham Grenville dyke SCG2 (CG06). (B) Comparison of our data and the similar directions reported by McCausland et al. (56) from the Chatham Grenville and Mont Rigaud stocks.

4.2 Temperature range and remanence carrier

A possible contribution from hematite has been inferred on the basis of remanence surviving above ~580 °C and the use of 590 °C in accepted Thellier results. In the Lloyd et al. (55) material, however, the maximum Curie temperature is ~580 °C, and the thermal demagnetiser had previously been shown on comparable material to produce offsets of approximately 10 °C owing to thermal hysteresis. SEM textures in these specimens are not diagnostic for distinguishing hematite from magnetite, and no independent evidence for hematite was identified. On the available evidence, there is therefore no positive basis for interpreting the accepted palaeointensity results as reflecting a hematite-held thermochemical remanence rather than a magnetite-dominated remanence. In addition, removing the 590°C step point would not notably change the result.

4.3 Thermal resetting within the baked-contact setting

Interpretation of the sampled dyke segment as thermally reset does not depend on the presence of diagnostic metamorphic mineral assemblages. It is supported primarily by palaeomagnetic

evidence: concordant specimen- and site-level ChRM directions between the dyke and the Early Cambrian intrusions, together with baked-contact observations showing that dykes sampled close to the intrusion carry the stock direction whereas more distal material preserves older magnetisations. Collectively, these observations support localised thermal resetting during emplacement of the stock. This conclusion is not weakened by the absence of obvious amphibolite, which can be difficult to recognise in narrow contact zones and may fail to develop diagnostic hornblende unless bulk chemistry is favourable (e.g., 59–61). Importantly, the difficulty of recognising amphibolite facies mineralisation in narrow contact zones is unrelated to the presence of abundant, datable hornblende in the larger intrusions targeted by $^{40}Ar/^{39}Ar$ geochronology.

4.4 Age of remanence acquisition

The cooling age constraints reported by McCausland et al. (56) are internally consistent and were obtained from multiple intrusions. Five hornblende $^{40}Ar/^{39}Ar$ plateau ages from MR10, CG31, and CG14 yield a weighted mean of 531.9 ± 1.4 Ma. Each plateau satisfies standard acceptance criteria, whereas disturbed K-feldspar spectra were explicitly excluded from the age interpretation (56). The ~10 – 15 Myr difference between these hornblende plateau ages and the slightly older zircon U–Pb ages cited by Zhou et al. (4) reflects closure-temperature differences rather than analytical error. Zircon U–Pb dating records crystallisation at temperatures above about 900 °C (62, 63), whereas hornblende $^{40}Ar/^{39}Ar$ dating records cooling through roughly 500 °C (64–66). Slow post-emplacement cooling, potentially combined with inherited zircon domains, provides a plausible explanation for the small offset.

Although inverse-isochron plots were not published by McCausland et al., (56), the concordant hornblende plateaus across multiple lithologic units argue strongly against significant excess argon. The available evidence therefore supports an Early Cambrian magnetisation age of ~532 Ma, fully consistent with our palaeomagnetic interpretation.

Although inverse-isochron plots were not published by McCausland et al. (56), the reproducibility of the hornblende plateau ages across multiple lithologic units suggests that substantial excess-argon effects are unlikely to dominate the dataset. The currently available geochronology does not resolve the exact timing of remanence acquisition with high precision. However, the available evidence remains more consistent with an Early Cambrian age for cooling and associated magnetisation than with any substantially older interpretation, and the hornblende plateau ages likely provide the more directly relevant constraint on remanence acquisition than zircon crystallisation ages.

4.5 Outcome for the comparative synthesis

Taken together, the directional, baked-contact, rock-magnetic, and geochronological evidence supports retention of the Lloyd et al. (55) whole-rock estimate in the comparative synthesis used here. The available data do not provide sufficient basis for excluding the estimate on grounds of unresolved lightning contamination, strong pTRM-tail bias, hematite-held thermochemcial remanence, or failure of thermal resetting. The estimate is therefore retained as an Early Cambrian weak-field determination, with the same general level of caution applied to all published palaeointensity data incorporated into the synthesis.

Supplementary Note 5. Detailed palaeointensity methods

Palaeointensity experiments targeted samples with well-defined ChRM directions and assessed over the relevant temperature intervals. Methods were chosen according to rock-magnetic behaviour; Bourinot volcanics were analysed using thermal Thellier (IZZI+), with multiple techniques applied to the Itabaiana dykes. All experiments were conducted within a zero-field environment.

5.1 Thermal and microwave Thellier methods

In thermal Thellier (IZZI+) experiments, alternating zero- and in-field heating pairs are integrated with pTRM checks to monitor alteration/multidomain (MD) effects (67–71). Intensities are taken from best-fit, origin-trending segments within the ChRM window Laboratory fields were 3-15 μT (Bourinot) and 10 μT (Itabaiana) with heating steps to 680 °C (Bourinot) and 590 °C (Itabaiana). Measurements were carried out on Rapid 2G and JR6 magnetometers.

The microwave protocol mirrors thermal Thellier (IZZI+), same (ZIIZP) sequence, Arai analysis, and checks, but uses short (~seconds) 14 GHz exposures that demagnetise/remagnetise the remanence carriers with minimal bulk heating, reducing thermochemical alteration (72–75). Laboratory fields (10 μT) were applied at angles between 45° and 135° to the ChRM so as to avoid the extremes of either suppressing or exaggerating zig-zagging behaviour (70).

5.2 Modified Shaw method

In modified Shaw (76–80) palaeointensity experiments, NRM is stepwise alternating field (AF) demagnetised before a laboratory TRM is imparted by a single heating (> T_C) and cooling in a known field, then AF-demagnetised over the same steps. After each step, the residual vector after the maximum AF is subtracted to match coercivity fractions. Pre/post-heating anhysteretic remanent magnetisation (ARM) ratios (ARM_0/ARM_1) correct for alteration to give TRM* (34, 35). Palaeointensity derives from the NRM-TRM* slope over a linear, origin-trending interval; a further heating (TRM_2) checks ARM corrections against the initial TRM (slopeT ≈ 1; 35, 36). This addition,

which is dependent on hold duration, is used here to avoid anomalous results rather than an overly strict criterion (80).

5.3 Wilson and Triaxe methods

The Wilson method (81) employs continuous measurements on a three-axis vibrating sample magnetometer (Triaxe-VSM from Mag-Instruments UG). A specimen is heated in zero field to ≥ T_C of the principal carrier. A single cooling in a known field then imparts a full TRM, followed by zero-field reheating to demagnetise that TRM. Palaeointensity is obtained from the NRM/TRM fraction ratio, with reversibility indicating minimal alteration (32) and quantified by the relative standard error (RSE) of the best-fit (82). The Triaxe method (32) uses five automated sequences between T_1 and T_2 (< T_C) chosen to avoid high-temperature alteration: M1 zero-field heat (NRM demag); M2-M3 reversible zero-field cool/heat (baseline, subtracted from M1/M5); M4 cool in B_{lab} set near parallel to the ChRM direction to acquire TRM; M5 zero-field re-heat (TRM demag). At each temperature, compute baseline-corrected NRM and TRM losses to obtain $\mathrm{R(T_i)}$ and $\mathrm{R'(T_i)}$; multiplied by B_{lab} to obtain the palaeointensity (32, 33).

5.4 Method selection and cross-method comparison

Palaeointensity experiments targeted samples with well-defined characteristic remanent magnetisations over appropriate temperature intervals. Methods were selected according to rock-magnetic behaviour, expected remanence stability, and signal strength. Thermal Thellier (IZZI+) remains the benchmark approach for samples displaying near-ideal behaviour. The microwave method follows the same protocol while minimising bulk heating, modified Shaw reduces repeated high-temperature exposure and, through use of full TRMs, can lessen multidomain effects, and the Wilson and Triaxe methods provide rapid continuous determinations and can reduce some effects of magnetic anisotropy. Agreement among methods therefore provides a strong internal check on the reliability of palaeointensity estimates.

These considerations led to different experimental strategies at the two localities. Bourinot samples were investigated using thermal Thellier experiments because they are weakly magnetised and carry a relatively high-coercivity remanence. Triaxe was not suitable for this material because of the weak signal and the high-temperature, high-coercivity component. Modified Shaw was also not appropriate because AF demagnetisation does not adequately separate the relevant remanence components, consistent with the original palaeomagnetic study (23). By contrast, Itabaiana samples had sufficient magnetisation and suitable behaviour for analysis using multiple complementary methods, including thermal and microwave Thellier, modified Shaw, and continuous Triaxe/Wilson approaches. This allowed direct comparison

among methods and provided additional checks on alteration, domain-state effects, and anisotropy-related bias.

Supplementary Tables

Supplementary Table S1. Determining Selection Criteria Optimised for Palaeointensity Experiments (SCOPE): literature compilation and threshold selection used to derive the SCOPE criteria.

Supplementary Table S2. Performance statistics for criteria sets applied to the calibration dataset, including accuracy, discrimination, and data-yield metrics.

Supplementary Table S3. Bourinot and Itabaiana specimen-level thermal and microwave Thellier palaeointensity results and associated selection statistics.

Supplementary Table S4. Shaw specimen results from this study.

Supplementary Table S5. Triaxe and Wilson method results from this study.

Supplementary Table S6. Anisotropy tensors for the anisotropy-corrected Shaw palaeointensity estimates.

Supplementary Table S7. Individual QPI scores for the new site means, with definitions of the component QPI criteria.

Supplementary Table S8. Specimen-level results from Zhou et al. (2024), including reported and recalculated values.

Supplementary Table S9. Specimen-level results from Zhou et al. (2022) under SCOPE, including recalculated values.

Supplementary Table S10. Specimen results from Bono et al. (2019) under SCOPE, including recalculated values.

Supplementary Table S11. Full list of selection criteria applied in this study, including the SCOPE thresholds and the corresponding criteria used for the other palaeointensity methods.

REFERENCES

1. W. Huang, J. A. Tarduno, T. Zhou, M. Ibañez-Mejia, L. Dal Olmo-Barbosa, E. Koester, E. G. Blackman, A. V. Smirnov, G. Ahrendt, R. D. Cottrell, K. P. Kodama, R. K. Bono, D. G. Sibeck, Y. X. Li, F. Nimmo, S. Xiao, M. K. Watkeys, Near-collapse of the geomagnetic field may have contributed to atmospheric oxygenation and animal radiation in the Ediacaran Period. *Commun. Earth Environ.* **5**, 207 (2024).

2. J. A. Tarduno, T. Zhou, W. Huang, J. Jodder, Earth's magnetic field and its relationship to the origin of life, evolution and planetary habitability. *Natl. Sci. Rev.* **12**, nwaf082 (2025).

3. T. Zhou, M. Ibañez-Mejia, R. K. Bono, R. D. Cottrell, W. Bleeker, K. P. Kodama, W. Huang, E. G. Blackman, F. Nimmo, A. V. Smirnov, J. A. Tarduno, Magnetization and age of ca. 544 Ma syenite, eastern Canada: Evidence for renewal of the geodynamo. *Earth Planet. Sci. Lett.* **639**, 118758 (2024).

4. T. Zhou, J. A. Tarduno, F. Nimmo, R. D. Cottrell, R. K. Bono, M. Ibanez-Mejia, W. Huang, M. Hamilton, K. Kodama, A. V. Smirnov, B. Crummins, F. Padgett, Early Cambrian renewal of the geodynamo and the origin of inner core structure. *Nat. Commun.* **13**, 4161 (2022).

5. V. V. Shcherbakova, V. G. Bakhmutov, D. Thallner, V. P. Shcherbakov, G. V. Zhidkov, A. J. Biggin, Ultra-low palaeointensities from East European Craton, Ukraine support a globally anomalous palaeomagnetic field in the Ediacaran. *Geophys. J. Int.* **220**, 1928–1946 (2020).

6. D. Thallner, A. J. Biggin, H. C. Halls, An extended period of extremely weak geomagnetic field suggested by palaeointensities from the Ediacaran Grenville dykes (SE Canada). *Earth Planet. Sci. Lett.* **568**, 117025 (2021).

7. D. Thallner, A. J. Biggin, P. J. A. McCausland, R. R. Fu, New paleointensities from the Skinner Cove Formation, Newfoundland, suggest a changing state of the geomagnetic field at the Ediacaran-Cambrian transition. *J. Geophys. Res. Solid Earth* **126**, e2021JB022292 (2021).

8. D. Thallner, V. V. Shcherbakova, V. G. Bakhmutov, V. P. Shcherbakov, G. V. Zhidkov, I. B. Poliachenko, A. J. Biggin, New palaeodirections and palaeointensity data from extensive

profiles through the Ediacaran section of the Volyn Basalt Province (NW Ukraine). *Geophys. J. Int.* **231**, 474–492 (2022).

9. R. K. Bono, J. A. Tarduno, F. Nimmo, R. D. Cottrell, Young inner core inferred from Ediacaran ultra-low geomagnetic field intensity. *Nat. Geosci.* **12**, 143–147 (2019).

10. F. T. Bowyer, A. J. Shore, R. A. Wood, L. J. Alcott, A. L. Thomas, I. B. Butler, A. Curtis, S. Hainanan, S. Curtis-Walcott, A. M. Penny, S. W. Poulton, Regional nutrient decrease drove redox stabilisation and metazoan diversification in the late Ediacaran Nama Group, Namibia. *Sci. Rep.* **10**, 2240 (2020).

11. G. E. Budd, S. Jensen, The origin of the animals and a 'Savannah' hypothesis for early bilaterian evolution. *Biol. Rev.* **92**, 446–473 (2017).

12. M. S. Dodd, W. Shi, C. Li, Z. Zhang, M. Cheng, H. Gu, D. S. Hardisty, S. J. Loyd, M. W. Wallace, A. vS. Hood, K. Lamothe, B. J. W. Mills, S. W. Poulton, T. W. Lyons, Uncovering the Ediacaran phosphorus cycle. *Nature* **618**, 974–980 (2023).

13. M. L. Droser, L. G. Tarhan, J. G. Gehling, The rise of animals in a changing environment: Global ecological innovation in the late Ediacaran. *Annu. Rev. Earth Planet. Sci.* **45**, 593–617 (2017).

14. W. P. Li, Y. Y. Zhao, M. Y. Zhao, X. P. Zha, Y. F. Zheng, Enhanced weathering as a trigger for the rise of atmospheric O_2 level from the late Ediacaran to the early Cambrian. *Sci. Rep.* **9**, 10630 (2019).

15. M. Lingam, Revisiting the biological ramifications of variations in Earth's magnetic field. *Astrophys. J. Lett.* **874**, L28 (2019).

16. J. Aubert, S. Labrosse, C. Poitou, Modelling the palaeo-evolution of the geodynamo. *Geophys. J. Int.* **179**, 1414–1428 (2009).

17. C. J. Davies, R. K. Bono, D. G. Meduri, J. Aubert, S. Greenwood, A. J. Biggin, Dynamo constraints on the long-term evolution of Earth's magnetic field strength. *Geophys. J. Int.* **228**, 316–336 (2021).

18. M. Landeau, J. Aubert, P. Olson, The signature of inner-core nucleation on the geodynamo. *Earth Planet. Sci. Lett.* **465**, 193–204 (2017).

19. P. E. Driscoll, Simulating 2 Ga of geodynamo history. *Geophys. Res. Lett.* **43**, 5680–5687 (2016).

20. C. Davies, M. Pozzo, D. Gubbins, D. Alfè, Constraints from material properties on the dynamics and evolution of Earth's core. *Nat. Geosci.* **8**, 678–685 (2015).

21. T. Nakagawa, S.-i. Takehiro, Y. Sasaki, Effect of thermal conductivity on the simultaneous formation of a stable region at the top of earth's core and magnetic field generation over four billion years. *Phys. Earth Planet. In.* **365**, 107380 (2025).

22. C. E. White, S. M. Barr, M. L. Bevier, S. Kamo, A revised interpretation of Cambrian and Ordovician rocks in the Bourinot belt of central Cape Breton Island, Nova Scotia. *Atlantic Geol.* **30**, 123–142 (1994).

23. R. Van der Voo, R. J. E. Johnson, Paleomagnetism of the Dunn Point Formation (Nova Scotia): High paleolatitudes for the Avalon Terrane in the Late Ordovician. *Geophys. Res. Lett.* **12**, 337–340 (1985).

24. R. I. F. Trindade, M. S. D'Agrella-Filho, I. Epof, B. B. Brito Neves, Paleomagnetism of Early Cambrian Itabaiana mafic dikes (NE Brazil) and the final assembly of Gondwana. *Earth Planet. Sci. Lett.* **244**, 361–377 (2006).

25. G. A. Paterson, L. Tauxe, A. J. Biggin, R. Shaar, L. C. Jonestrask, On improving the selection of Thellier-type paleointensity data. *Geochem. Geophys. Geosyst.* **15**, 1180–1192 (2014).

26. B. Cych, M. Morzfeld, L. Tauxe, Bias Corrected Estimation of Paleointensity (BiCEP): An improved methodology for obtaining paleointensity estimates. *Geochem. Geophys. Geosyst.* **22**, e2021GC009755 (2021).

27. A. W. Tully, G. A. Paterson, Another simple test for the presence of multidomain behavior during paleointensity experiments. *J. Geophys. Res. Solid Earth* **130**, e2025JB031608 (2025).

28. R. Day, M. Fuller, V. A. Schmidt, Hysteresis properties of titanomagnetites: Grain-size and compositional dependence. *Phys. Earth Planet. In.* **13**, 260–267 (1977).

29. W. Williams, R. Moreno, A. R. Muxworthy, G. A. Paterson, L. Nagy, L. Tauxe, U. Donardelli Bellon, A. A. Cowan, I. Ferreira, Vortex magnetic domain state behavior in the day plot. *Geochem. Geophys. Geosyst.* **25**, e2024GC011462 (2024).

30. R. Shaar, H. Ron, L. Tauxe, R. Kessel, A. Agnon, Paleomagnetic field intensity derived from non-SD: Testing the Thellier IZZI technique on MD slag and a new bootstrap procedure. *Earth Planet. Sci. Lett.* **310**, 213–224 (2011).

31. B. Cych, L. Tauxe, G. Cromwell, J. Sinton, A. A. P. Koppers, Changes in non-dipolar field structure over the Plio–Pleistocene: New paleointensity results from Hawai'i compared to global data sets. *J. Geophys. Res. Solid Earth* **128**, e2023JB026492 (2023).

32. M. Le Goff, Y. Gallet, A new three-axis vibrating sample magnetometer for continuous high-temperature magnetization measurements: Applications to paleo- and archeo-intensity determinations. *Earth Planet. Sci. Lett.* **229**, 31–43 (2004).

33. Y. Gallet, M. Le Goff, A. Genevey, Triaxe archeointensity analysis. *Phys. Earth Planet. In.* **332**, 106924 (2022).

34. A. J. Biggin, G. A. Paterson, A new set of qualitative reliability criteria to aid inferences on palaeomagnetic dipole moment variations through geological time. *Front. Earth Sci.* **2**, 24 (2014).

35. M. R. Koymans, D. J. J. van Hinsbergen, D. Pastor-Galán, B. Vaes, C. G. Langereis, Towards FAIR paleomagnetic data management through Paleomagnetism.org 2.0. *Geochem. Geophys. Geosyst.* **21**, e2019GC008838 (2020).

36. A. Béguin, G. A. Paterson, A. J. Biggin, L. V. de Groot, Paleointensity.org: An online, open source, application for the interpretation of paleointensity data. *Geochem. Geophys. Geosyst.* **21**, e2019GC008791 (2020).

37. R. K. Bono, G. A. Paterson, A. van der Boon, Y. A. Engbers, J. Michael Grappone, B. Handford, L. M. A. Hawkins, S. J. Lloyd, C. J. Sprain, D. Thallner, A. J. Biggin, The PINT database: A definitive compilation of absolute palaeomagnetic intensity determinations since 4 billion years ago. *Geophys. J. Int.* **229**, 522–545 (2022).

38. L. Tauxe, J. S. Gee, M. B. Steiner, H. Staudigel, Paleointensity results from the Jurassic: New constraints from submarine basaltic glasses of ODP Site 801C. *Geochem. Geophys. Geosyst.* **14**, 4718–4733 (2013).

39. T. H. Torsvik, L. R. M. Cocks, *Earth History and Palaeogeography* (Cambridge Univ. Press, 2017).

40. N. M. Levashova, I. V. Golovanova, D. V. Rudko, K. N. Danukalov, S. V. Rudko, S. R. Yu, J. G. Meert, Late Ediacaran magnetic field hyperactivity: Quantifying the reversal frequency in the Zigan Formation, Southern Urals, Russia. *Gondw. Res.* **94**, 133–142 (2021).

41. Y. Gallet, V. Pavlov, I. Korovnikov, Extreme geomagnetic reversal frequency during the Middle Cambrian as revealed by the magnetostratigraphy of the Khorbusuonka section (northeastern Siberia). *Earth Planet. Sci. Lett.* **528**, 115823 (2019).

42. F. Shen, B. Wen, P. J. A. McCausland, Z. Gong, Z. Zhu, F. Liu, J. Wang, A >70-Myr-long geomagnetic field reversal hyperactivity across the Ediacaran-Cambrian transition. *Geophys. Res. Lett.* **52**, e2025GL118030 (2025).

43. S. E. Haggerty, Oxide textures—A mini-atlas. *Rev. Mineral. Geochem.* **25**, 129–219 (1991).

44. P. A. Selkin, L. Tauxe, Long-term variations in palaeointensity. *Philos. Trans. R. Soc. London Ser. Math. Phys. Eng. Sci.* **358**, 1065–1088 (2000).

45. Y. Usui, N. Nakamura, Nonlinear thermoremanence corrections for Thellier paleointensity experiments on single plagioclase crystals with exsolved magnetites: A case study for the Cretaceous Normal Superchron. *Earth Planets Space* **61**, 1327–1337 (2009).

46. C. Kato, M. Sato, Y. Yamamoto, H. Tsunakawa, J. L. Kirschvink, Paleomagnetic studies on single crystals separated from the middle Cretaceous Iritono granite. *Earth Planets Space* **70**, 176 (2018).

47. O. Ageeva, G. Habler, S. A. Gilder, R. Schuster, A. Pertsev, O. Pilipenko, G. Bian, R. Abart, Oriented magnetite inclusions in plagioclase: Implications for the anisotropy of magnetic remanence. *Geochem. Geophys. Geosyst.* **23**, e2021GC010272 (2022).

48. B. Cych, G. A. Paterson, L. Nagy, W. Williams, B. Moskowitz, Magnetic domain states and critical sizes in the titanomagnetite series. *J. Geophys. Res. Solid Earth* **129**, e2024JB028805 (2024).

49. G. A. Paterson, A simple test for the presence of multidomain behavior during paleointensity experiments. *J. Geophys. Res. Solid Earth* **116**, B10104 (2011).

50. M. H. Dodson, E. McLelland-Brown, Magnetic blocking temperatures of single-domain grains during slow cooling. *J. Geophys. Res.* **85**, 2625–2637 (1980).

51. A. Ferk, R. Leonhardt, K. U. Hess, S. Koch, R. Egli, D. Krása, D. B. Dingwell, Influence of cooling rate on thermoremanence of magnetite grains: Identifying the role of different magnetic domain states. *J. Geophys. Res. Solid Earth* **119**, 1599–1606 (2014).

52. A. J. Biggin, S. Badejo, A. R. Muxworthy, M. J. Dekkers, The effect of cooling rate on the intensity of thermoremanent magnetization (TRM) acquired by assemblages of pseudo-single domain, multidomain and interacting single-domain grains. *Geophys. J. Int.* **193**, 1239–1249 (2013).

53. S. L. Halgedahl, R. D. Jarrard, Low-temperature behavior of single-domain through multidomain magnetite. *Earth Planet Sci. Lett.* **130**, 127–139 (1995).

54. R. K. Bono, J. A. Tarduno, A stable Ediacaran Earth recorded by single silicate crystals of the ca. 565 Ma Sept-Îles intrusion. *Geology* **43**, 131–134 (2015).

55. S. J. Lloyd, A. J. Biggin, G. A. Paterson, P. J. A. McCausland, Extremely weak early Cambrian dipole moment similar to Ediacaran: Evidence for long-term trends in geomagnetic field behaviour? *Earth Planet. Sci. Lett.* **595**, 117757 (2022).

56. P. J. A. McCausland, R. Van der Voo, C. M. Hall, Circum-Iapetus paleogeography of the Precambrian-Cambrian transition with a new paleomagnetic constraint from Laurentia. *Precambrian Res.* **156**, 125–152 (2007).

57. G. S. Watson, A test for randomness of directions. *Geophys. J. Int.* **7**, 160–161 (1956b).

58. L. Tauxe, *Essentials of Paleomagnetism, 5th web ed.* (University of California Press, 2021).

59. B. W. D. Yardley, *An Introduction to Metamorphic Petrology*, Longman Earth Science Series (Longman Scientific & Technical, 1989).

60. J. D. Winter, *Principles of Igneous and Metamorphic Petrology (2nd ed.)* (Pearson/Prentice Hall, 2010).

61. F. S. Spear, *Metamorphic Phase Equilibria and Pressure–Temperature–Time Paths (Monograph 1)* (Mineralogical Society of America, 1993).

62. T. M. Harrison, E. B. Watson, Kinetics of zircon dissolution and zirconium diffusion in granitic melts of variable water content. *Contrib. Mineral. Petrol.* **84**, 66–72 (1983).

63. D. J. Cherniak, E. B. Watson, Pb diffusion in zircon. *Chem. Geol.* **172**, 5–24 (2000).

64. T. M. Harrison, I. McDougall, Investigations of an intrusive contact, northwest Nelson, New Zealand—I. Thermal, chronological, and isotopic constraints. *Geochim. Cosmochim. Acta* **44**, 1985–2003 (1980).

65. T. M. Harrison, Diffusion of ^{40}Ar in hornblende. *Contrib. Mineral. Petrol.* **78**, 324–331 (1982).

66. I. M. Villa, Isotopic closure. *Terra Nova* **10**, 42–47 (1998).

67. E. Thellier, O. Thellier, Sur l'intensité du champ magnétique terrestre dans le passé historique et géologique. *Ann. Géophys.* **15**, 285–376 (1959).

68. R. S. Coe, Paleo-intensities of the Earth's magnetic field determined from Tertiary and Quaternary rocks. *J. Geophys. Res.* **72**, 3247–3262 (1967).

69. M. J. Aitken, A. L. Allsop, G. D. Bussell, M. B. Winter, Determination of the intensity of the Earth's magnetic field during archaeological times: Reliability of the Thellier technique. *Rev. Geophys.* **26**, 3–12 (1988).

70. Y. Yu, L. Tauxe, Testing the IZZI protocol of geomagnetic field intensity determination. *Geochem. Geophys. Geosyst.* **6**, Q05H17 (2005).

71. L. Tauxe, H. Staudigel, Strength of the geomagnetic field in the cretaceous normal superchron: New data from submarine basaltic glass of the troodos ophiolite. *Geochem. Geophys. Geosyst.* **5**, Q02H06 (2004).

72. D. Walton, J. Shaw, J. Share, J. Hakes, Microwave demagnetization. *J. Appl. Phys.* **71**, 1549–1551 (1992).

73. H. Böhnel, A. J. Biggin, D. Walton, J. Shaw, J. A. Share, Microwave palaeointensities from a recent Mexican lava flow, baked sediments and reheated pottery. *Earth Planet. Sci. Lett.* **214**, 221–236 (2003).

74. N. Suttie, J. Shaw, M. Hill, Direct demonstration of microwave demagnetization of a whole rock sample with minimal heating. *Earth Planet. Sci. Lett.* **292**, 357–362 (2010).

75. S. Lloyd, A. Biggin, M. Hill, L. De Groot, N. Suttie, J. Morris, H. Boehnel, J. Shaw, The use of high frequency microwaves in absolute palaeomagnetic intensity experiments. *Front. Earth Sci.* **11**, 1188528 (2023).

76. J. Shaw, A new method of determining the magnitude of the palaeomagnetic field: Application to five historic lavas and five archaeological samples. *Geophys. J. R. Astron. Soc.* **39**, 133–141 (1974).

77. T. C. Rolph, J. Shaw, A new method of palaeofield magnitude correction for thermally altered samples and its application to Lower Carboniferous lavas. *Geophys. J. Int.* **80**, 773–781 (1985).

78. Y. Yamamoto, H. Tsunakawa, H. Shibuya, Palaeointensity study of the Hawaiian 1960 lava: Implications for possible causes of erroneously high intensities. *Geophys. J. Int.* **153**, 263–276 (2003).

79. H. Tsunakawa, J. Shaw, The Shaw method of palaeointensity determinations and its application to recent volcanic rocks. *Geophys. J. Int.* **118**, 781–787 (1994).

80. S. Lloyd, G. A. Paterson, D. Thallner, A. J. Biggin, Improvements to the Shaw-type absolute palaeointensity method. *Front. Earth Sci.* **9**, 701863 (2021).

81. R. L. Wilson, Palaeomagnetism in Northern Ireland. Part I: The thermal demagnetization of natural magnetic moments in rocks. *Geophys. J. Int.* **5**, 45–58 (1961).

82. A. R. Muxworthy, Revisiting a domain-state independent method of palaeointensity determination. *Phys. Earth Planet. In.* **179**, 21–31 (2010).
